# Lecture on Pleurisy,—Gangrene of the Lungs,—Meningitis,—Pneumonia,—Rheumatism,—Pathological Anatomy of Pleurisy

**Published:** 1840-01-25

**Authors:** W. W. Gerhard


### CLINICAL LECTURE.

### PHILADELPHIA HOSPITAL.

*Wednesday, January 15th*, 1840.

**LECTURE ON PLEURISY,—GANGRENE OF THE LUNGS,—MENINGITIS,—PNEUMONIA,—RHEUMATISM,—PATHOLOGICAL ANATOMY OF PLEURISY.**

By W. W. Gerhard, M. D.

*No.* 9—*Winter Course.*

I will commence to-day by showing you the progress of certain cases of disease which have already been before you; and then introduce to you some cases of rheumatism, concluding the lecture with an examination of the lungs of an individual who died of pleurisy.

*Case* 1.—This man you saw last week. He was labouring under a complication of pleurisy and pneumonia, the former being the predominant disease. He is now convalescent, but some of the signs of the disease still remain. The most obvious of these is an enlargement of the right side of the chest. This, in most cases of pleurisy, is found persisting after the cessation of the general symptoms; and various other local signs not unfrequently continue along with it. The enlargement does not subside as soon as the inflammation, because time is required for the absorption of the effused matter; this time being greater or less according to the quality and nature of the effusion. The enlargement of the chest, from the continuance of the effusion, may be detected either by the eye, or by actual measurement. I generally ascertain it by the eye alone: this is the most convenient and easy method, when the eye has been somewhat practised in the observation of such matters. As you perceive, the effusion is likewise indicated by dulness on percussion, which, however, has become slight, and is almost confined to the base of the lung. The liquid thus gradually disappearing, the pressure is removed from the lung, and the vesicular character of the respiration is almost entirely restored. Along with these physical signs, one striking symptom of the disease only remains. This is dyspnœa: it is not constant, but is produced by any considerable exertion, such as ascending a flight of stairs. In this, as in most other cases of convalescence from pleurisy, there also remains a preternatural susceptibility to the impression of cold. In many instances there is likewise a sense of pricking or slight pain in the chest, experienced when the patient takes a full inspiration, or makes any exertion which requires a considerable distention of the chest. This symptom is owing to the stretching of the recent adhesions, which is produced by the motion. These adhesions are very sensible during the first stages of their organization; but when this process is completed, and the false membrane assumes the ordinary characters of serous membranes, this sensibility disappears, and motion produces no pain. A Burgundy pitch plaster has been applied to the chest of this patient, with a double object. In the first place, it is calculated to prevent too great motion of the pleuræ against each other, in the same manner that adhesive strips act when applied over a fractured rib. Secondly, it weakens the impression of cold upon the chest; and then, again, it is a good, permanent counter-irritant. For some time past no general treatment has been necessary.

*Case* 2.—This patient has also been before you more than once. He has been labouring under gangrene of the lungs consequent upon pneumonia. He is now convalescent, and is somewhat in the condition of the patient whom you have just seen; that is, he suffers from the consequences of disease, rather than from disease itself, the active signs of the latter having entirely ceased. The man, as you see, walks about without any difficulty; there is still some cough, more particularly in the morning; the expectoration is muco-purulent, and has lost its gangrenous appearance; the appetite is better, and the strength rapidly improving; the peculiar expression of his countenance is likewise indicative of convalescence. This change in the expression of the countenance, in convalescence, from acute disease, has not, I think, been sufficiently attended to by authors; it is a sort of subsidence of the features. Thus, at the decline of fevers and other acute diseases, we often observe a sudden paleness, accompanied by a sinking of the pulse, which sometimes falls, even in children, as low as fifty or sixty in the minute, and at the same time is often irregular. This state may be called the subsidence after fever, and is one of the best signs of convalescence.

For some days it has been unnecessary to put the patient under any kind of treatment, except the use of remedies calculated to relieve the few unpleasant symptoms which remain. During the progress of the pneumonia, and more particularly of the subsequent gangrene, it was necessary to employ stimuli, in order to support the system. These have been discontinued, and the patient has been put on the use of a combination of syrup of tolu, tartar emetic, and laudanum, for the relief of the cough. Opiates are extremely useful as a palliation of cough; but in acute pulmonary diseases, when there is much dyspnœa and general excitement, they must be used very cautiously. In such a case as the present, of course there can be no objection to their use.

*Case* 3.—This patient, whom you saw sometime ago, is also convalescent. At that time he was labouring under meningitis, which was well characterized by the ordinary signs, viz., delirium during the night, alternating with restlessness during the day; disturbance of the functions of motility and sensibility; contraction of the pupils; distortion of the mouth, and more or less rigidity of the extremities. The latter signs were not confined to one side of the body, but shifted from one to the other; they were well marked and permanent. If the delirium and other signs had continued only one or two days, they would have indicated merely functional disorder of the brain; but their continuance for several days in succession, rendered the existence of meningitis certain. All the signs of meningitis which existed in the present case have gradually declined, and the patient is now in a state of complete convalescence. The case is interesting, from the completeness of the cure which has been effected: this disease is, however, extremely subject to relapses.

*Case* 4.—At one of the preceding lectures I introduced a man named Carpenter, who was labouring under a complication of pleurisy and pneumonia, with probable tubercles. He is so ill at present, that it is impossible to bring him before you; and I shall, therefore, only explain to you the changes that have taken place. When you saw him he was doing well; and I was induced, from this favourable aspect of the case, to predict a speedy recovery. But he has since had an exacerbation of symptoms. The pneumonic symptoms had considerably subsided; the relapse was owing to a sudden increase of the pleuritic effusion, in consequence either of cold, or of the epidemic constitution, which, at present, seems to predispose to inflammation of serous membranes. The increased effusion caused a corresponding pressure on the lung, which was already inflamed; and hence arose an extreme degree of dyspnœa and prostration, so that for some days past the patient's condition has been very critical. His danger has been increased by the occurrence of endocarditis. In such a state of things, the treatment becomes a matter of great nicety and caution. The condition of the system obviously forbids large abstractions of blood. The only depletion which I have ventured to practise, was the application of a single cup to the right side, to be repeated or not, according to circumstances. This may seem trifling to those who have been accustomed only to heroic bleedings in internal inflammations. But we should reflect that our object in bleeding is only to assist the operations of nature. In certain cases, by too large depletion, we are in danger of totally defeating them, and hastening the prostration of the system. After the second application of the cup, there was a very manifest improvement, as evinced by the great diminution of the dyspnœa. Even a single application is often of great service in pneumonia and pleurisy. I think the application of a single cup, repeated according to the urgency of the symptoms, preferable to venesection, or to the application of many cups, in certain cases of these diseases. But what, you may ask, are these cases? Exactly those in which the strength of the patient is exhausted by the continuance of the present disease, or by some previous one, when the powers of the system and the disease are nicely balanced. The single cup, which will take two or three ounces of blood, is a feeler, as it were, and you may afterwards, if advisable, cup again and again, or bleed. Don't misunderstand me, however; I do not wish to overrate the danger of large bleeding, or to discourage you from resorting to this powerful remedy in many cases. I wish only to let you know how much careful management of our therapeutic agents will do, especially at the nicely balanced period of the disease. I do not care to recommend very free depletion; to this, like all young practitioners, you will, I dare say, be ready enough to resort: I only warn you of the cases where this mode of treatment is not immediately necessary, nor proper, whatever it may be afterwards. I am confident that I have seen many cases of inflammatory diseases brought to a fatal termination by the incautious abstraction of blood.

Mercury has also been employed in this case, with the design of inducing slight ptyalism, and along with it the antiphlogistic action of the remedy: the ptyalism is not intended as a mode of depletion, but as a mark of the constitutional influence of mercury. This article is an admirable remedy in certain cases of acute inflammation; but this very utility has led to its abuse, and a consequent prejudice against it. It should always be recollected, that in employing mercury, we are not to push it to the extent of actual salivation; it ought to be suspended the moment it produces a slight redness of the gums, or other mark of its constitutional action. When such marks are evident, we almost always observe an immediate decline of the inflammatory action. By this cautious use of mercury, we may obtain all its good effects, without incurring the blame arising from the practice of pushing it to an injudicious extent. Under the moderate use of mercury, and very cautious depletion, our patient is again considerably improved.

I shall next show you a few cases of rheumatism. This disease is closely connected with inflammation of serous membranes, and I therefore usually consider them at the same time, or in succession. This connexion of these diseases is twofold. 1. In most cases of rheumatism, the synovial membrane of the joint is inflamed. 2. The internal serous membranes are affected by sympathy with the external ones. It was once supposed that when the internal serous membranes were affected, there was a metastasis of the inflammation; that is, the rheumatism was supposed to cease before the internal disease commenced. This is now known to be an error. We frequently observe pericarditis or endocarditis, occurring during an attack of rheumatism, reach its greatest degree of severity precisely at the time that the rheumatism is most severe. Cases in which metastasis really occurs, are exceptions to the general rule.

*Case* 1.—The patient, aged forty-five, was admitted January 10th. He was a sailor until two years ago; since which time, he has been engaged about the wharves of this city as a "stevedore." He has always enjoyed good health, with the exception of an attack of fever in Calcutta, in the year 1811. About two years since he began to suffer pains in the legs and lumbar region, which were aggravated by damp and cold weather. They have lately become more constant and severe. He has been bled, and has taken medicine for their relief, but without benefit. On the 10th he complained of acute pain in the back, hips, and knees, attended by fever, and great difficulty of locomotion. Dover's powder was ordered. 11*th*.—Much better. Six dry cups were applied to the lumbar region, which were followed by great relief. 12*th*.—Pain in loins entirely gone; elsewhere not diminished. 13*th*.—Still improving: action of heart perfectly natural, as to sound and impulse. Six cups applied to lumbar region, which took nine ounces of blood. The pains in every part were much diminished by the bleeding. But the patient still complained of costiveness, and sweating at night. Rhubarb and Dover's powder were ordered.

This is a case of the subacute grade of rheumatism. There has been little or no swelling, but much pain in the lumbar region, and in the joints of the lower extremities. During the last two months the pain in the latter parts has been confined to the left side. Acute rheumatism exhibits a marked difference in the symptoms. There is not only severe pain in the joints, but a decided swelling, with a more or less distinct blush on the surface. These symptoms arise from inflammation of the fibrous membranes about the joint, extending also to the synovial membrane, and followed by effusion into its cavity. The inflammation often leaves one joint, and appears in another; in the one first attacked it gradually declines, and when it has almost disappeared, pain and swelling are developed in the other joint. Thus, the disease is transferred from joint to joint, until at last five or six may become implicated in the disease. The articulations most frequently affected are those of the wrist, elbow, knee, and ankle; the shoulder and hip are much less commonly the seat of rheumatism. Along with these local signs, the general symptoms of excitement are likewise very well marked. The pulse, particularly, is excited to a degree that may be termed extraordinary. It has a fulness and resistance which are hardly met with in any other form of disease. This variety of pulse is the most striking characteristic of the *rheumatic fever.* The heart is more or less affected in a very large proportion of cases. Sometimes its disorder is merely functional; there is only an increase of its action. But in at least one-half of the cases of severe inflammatory rheumatism, there is actual disease—an inflammation of its investing and lining membranes—though either of them may be affected singly. Strange as it may appear, very few die of these acute inflammations of the heart. Why, therefore, is attention to them so important? It is because they are apt to result in a permanent organic lesion, from which the patient's life will be really in danger. Hence the physician should, in all cases of acute rheumatism, be on the look-out for the signs of cardiac disease; and, when detected, they should receive the most active attention. Generally, such affections will be manifested by a loud rasping or bellows sound, and dulness on percussion.

With regard to the treatment of inflammatory rheumatism, I shall, at present, say nothing, further than to observe that the natural course of the disease, when fairly begun, cannot be arrested abruptly by any means within our power. Various plans have been devised for the accomplishment of this end, but none of them have been found to succeed with certainty. Notwithstanding all our efforts, the disease will frequently continue for several weeks or months without the slightest abatement.

In subacute rheumatism, which more immediately interests us, Dover's powder is one of the best remedies at our command. It has been used in the case last detailed with the most marked advantage. It acts both as a diaphoretic and an anodyne. Sweating will not cure the disease; it is, in fact, one of its invariable symptoms, unless prevented from taking place by some accidental circumstance, as a cold room, &c. When this is the case, the patient's sufferings are aggravated; hence sweating appears to be a natural palliative of the disease, and remedies calculated to encourage it, cannot but do good, though they may not effect a cure. In some inflammatory cases, sweating appears to be more properly a curative process, almost a crisis. Dry cups were also applied to the loins in the preceding case, and acted very well. Scarification was not at first directed, in order that the action of the Dover's powder might not be interfered with. Two days afterwards the cups were repeated, but this time nine ounces of blood were drawn. A still greater improvement followed this application, and the patient has ever since continued convalescent.

You will remark that the local treatment in this case was directed to the spine alone. This method of treatment has been lately introduced by some English surgeons, especially Messrs. Teal and Tate, in neuralgia. Dr. Mitchell was the first, at least in this country, who adopted this practice in rheumatism, as well as neuralgia. He applies it to acute, as well as other forms of rheumatism. My own experience has convinced me that it is best adapted to subacute cases, in which the spine and large joints of the extremities are simultaneously affected; and the pains radiate from the spine towards the limbs, especially if increased on pressure: in such instances, cups to the affected portion of the spine act almost as a specific. The utility of the practice is illustrated by the case under consideration. The first effect of cupping was to diminish the pain in the loins; but the knee and hip were subsequently relieved without any special application of the treatment to them. In some cases, however, the pain in the extremities continues after the subsidence of that in the spine; and then, of course, the cups have to be applied to the joints themselves. However perfect the cure may be, a return of the disease is nevertheless always to be looked for in rheumatic affections.

*Case* 2.—This patient has been affected with intermittent neuralgic pains connected with disease of the spine. He was before the class about the commencement of the course, and then presented two swellings over the dorsal vertebræ, which I considered dependent on disease of the bodies of the vertebræ. This affection was treated with moxas, cupping, rest, and occasional purging. Under this treatment the swellings have in a great measure subsided; the paralysis of the lower extremities, which accompanied them, has disappeared; and the patient can walk about without inconvenience. But some signs of the disease still remain. A few days since the patient complained of pain at the outer part of the thighs: it commenced about 4 o'clock in the afternoon, and continued an hour and a half. It returned the next day at the same hour, and continued for the same length of time. Thus, it continued in an intermittent course for seven or eight days. To test the antiperiodic powers of quinine, six grains of this article were then directed, to be taken in three doses. The pain has not returned since. You will perceive, therefore, that rheumatism, connected with affections of the spine, requires a different treatment, according to its character as regards permanence or periodicity. In the former case it is treated by cups, Dover's powder, &c.; in the latter, the sulphate of quinine is the best remedy that can be employed. It operates in the same manner that it does in intermittent fever; and, as you well know, intermittent neuralgia is of the same essential nature with intermittent fever, and is indeed frequently a consequence of it.

*Case 3d.*—This man was taken in the latter part of December with chills, followed by fever, and pains in the joints. On the 6th of January, (at which time he entered the hospital,) he complained of pain in the left shoulder and elbow, the hips, and the right ankle; sweating at night, and costiveness; the action of the heart was natural; there was little fever; no swelling of the joints. The case appears to possess more of a neuralgic, than of an inflammatory character; in proper inflammation of the joints, there is always more or less effusion into their cavities. This distinction must always be observed, for there is a corresponding discrepancy in the nature and success of the treatment adapted to the two forms of disease. This subacute or neuralgic rheumatism is very easily cured in most instances, while true inflammation of the joints is apt to prove extremely obstinate. The treatment which has been adopted in this case is as follows: On the 6th, Dover's powders and the tincture of cimicifuga, or actæa racemosa, were ordered. A gradual improvement followed the use of these remedies. On the 11th, the patient had taken 150 drops of the tincture of cimicifuga, with a constant amelioration of the symptoms. Both the Dover's powders and the cimicifuga have doubtless contributed to the cure, but most good has probably been derived from the former. When Dover's powder is employed at the same time with cups to the affected part, as in one of the cases already noticed, we are able in a great measure, to analyse their operation, and judge of the effects of each. But here there has been a gradual decline of the symptoms in all the joints affected, and it is impossible to decide upon the relative influence exercised by the two remedies in the production of this result. In order to test more accurately the powers of the actæa racemosa, it is my intention to give it an exclusive trial in some cases of rheumatism of this variety.

I shall conclude the lecture by the examination of a pathological specimen. The lung before us, was taken from a subject who died of disease of the chest complicated with epilepsy. For particular reasons, the brain was not examined. The characters presented by the lung on dissection, as well as the symptoms during life, indicated the existence of a very aggravated degree of pleurisy, accompanied by pneumonia, affecting the left side. After the disease had continued some days, the pneumonia was considerably diminished; but the pleurisy grew suddenly worse, and this unfavourable change was accompanied by the development of an intercurrent pneumonia of the right side. The symptoms consequent upon this state of things, were still further aggravated by the disorder of the brain, which increased the difficulty of breathing, and diminised the power of expectoration. Under this complication of unfavourable circumstances, the patient died. When the muscular power necessary for efficient expectoration has been weakened either by old age or disorder of the brain, patients may suddenly expire on making any exertion, such as rising up in bed, owing to the matter in the bronchial tubes of the affected side rising towards the trachea, and flowing into the bronchiæ of the healthy side; being unable to expectorate the matter, the patients die from suffocation. This circumstance is apt to occur in asthenic pneumonia, and more particularly in peripneumonia notha.

Here is the lung which exhibits the marks of pleurisy. A part of the effusion still remains upon the surface of the pleura, viz ; the lymph. When the chest was opened, the lung was surrounded by a copious effusion of serum. In very protracted cases, the effusion becomes more or less purulent. The lymph is seen covering both the costal and pulmonary pleuræ in amorphous masses, which are still soft and without any traces of organization. If the pleurisy had been more prolonged, these masses of lymph would have been firm, and in the course of organization. When this process commences, the lymph becomes more firm, and globules of blood are seen at different points of the plastic mass, which becomes more and more numerous, and by running together form vessels ; these vessels finally become connected with those of the adjacent serous membrane. The lymph is thus converted into an organized membrane, by a process similar to that which takes place in the incubated egg. But the series of changes do not stop here. The vessels of the newly formed membrane gradually cease to carry red blood, it becomes transparent, and if sufficiently to allow motion, it finally presents all the characters of the natural serous membrane. As the lymph when first effused is adhesive, it attaches itself both to the pulmonary and costal pleuræ, so that when completely organized, it becomes a permanent bond of union between these two surfaces. The exact form of these adhesions will depend upon their extent ; if small, they assume in the most perfect degree, the appearance of serous membranes, but if they cover a considerable portion of the lung, they adhere so closely as to allow no motion, and possess more of the characters of cellular tissue. If inflammation of the pleura be moderate and of no very protracted continuance, no thickening of the membrane results ; but if long continued, a certain degree of thickening is invariably observed: in this case it has not occurred. The lung was greatly compressed by the effusion, and contains little air. Some marks of pneumonia are perceptible ; but pneumonia cannot occur in a very marked degree, where there is much pleuritic effusion ; for the pressure prevents the development of inflammation in the lungs, on the same principle that mechanical compression is employed in the treatment of external inflammations.

In the right lung we have the evidences of the pneumonia, which was the immediate cause of the patient's death. It is confined to the root of the lungs, and has not advaned beyond the second stage. The pulmonary tissue offers a less granulated appearance than usual ; when pressure is made, a thick mucus exudes. The obstruction of the tubes which resulted from the accumulation of this secretion, produced a fatal oppression, as the opposite lung, owing to the pressure of the surrounding fluid, was rendered incapable of performing its office. On the surface of the pleura is a perfectly organized band of serous membrane, indicating the existence of a pleurisy at some former period of the patient's life.

